# A novel aging-associated lncRNA signature for predicting prognosis in osteosarcoma

**DOI:** 10.1038/s41598-024-51732-1

**Published:** 2024-01-16

**Authors:** Yi He, Xiao Huang, Yajie Ma, Guohui Yang, Yuqing Cui, Xuefeng Lv, Rongling Zhao, Huifang Jin, Yalin Tong, Xinyu Zhang, Jitian Li, Mengle Peng

**Affiliations:** 1 Department of Mini-Invasive Spinal Surgery, The Third People’s Hospital of Henan Province, Zhengzhou, 450006 Henan China; 2 Department of Clinical Laboratory, Luohe Central Hospital, Luohe, 462300 Henan China; 3 Department of Medical Affair, The Third People’s Hospital of Henan Province, Zhengzhou, 450006 Henan China; 4 Department of Emergency Surgery, The First Affiliated Hospital of Zhengzhou University, Zhengzhou, 450052 Henan China; 5 General ICU, The First Affiliated Hospital of Zhengzhou University, Zhengzhou, 450052 Henan China; 6 Department of Clinical Laboratory, The Third Affiliated Hospital of Zhengzhou University, Zhengzhou, 450052 Henan China; 7 Department of Clinical Laboratory, The Third People’s Hospital of Henan Province, Zhengzhou, 450006 Henan China; 8 Department of Blood Transfusion, The First Affiliated Hospital of Zhengzhou University, Zhengzhou, 450052 Henan China; 9 Department of Digestion, The First Affiliated Hospital of Zhengzhou University, Zhengzhou, 450052 Henan China; 10 Henan Luoyang Orthopedic Hospital (Henan Provincial Orthopedic Hospital), Henan Provincial Orthopedic Institute, Henan University of Chinese Medicine, 100 Yongping Road, Zhengzhou, 450000 Henan China

**Keywords:** Cancer, Biomarkers, Oncology, Pathogenesis, Risk factors

## Abstract

Osteosarcoma (OS) is one of the most prevalent bone tumors in adolescents, and the correlation between aging and OS remains unclear. Currently, few accurate and reliable biomarkers have been determined for OS prognosis. To address this issue, we carried out a detailed bioinformatics analysis based on OS with data from the Cancer Genome Atlas data portal and Human Aging Genomic Resources database, as well as in vitro experiments. A total of 88 OS samples with gene expression profiles and corresponding clinical characteristics were obtained. Through univariate Cox regression analysis and survival analysis, 10 aging-associated survival lncRNAs (AASRs) were identified to be associated with the overall survival of OS patients. Based on the expression levels of the 10 AASRs, the OS patients were classified into two clusters (Cluster A and Cluster B). Cluster A had a worse prognosis, while Cluster B had a better prognosis. Then, 5 AASRs were ultimately included in the signature through least absolute shrinkage and selection operator-Cox regression analysis. Kaplan‒Meier survival analysis verified that the high-risk group exhibited a worse prognosis than the low-risk group. Furthermore, univariate and multivariate Cox regression analyses confirmed that the riskScore was an independent prognostic factor for OS patients. Subsequently, we discovered that the risk signature was correlated with the properties of the tumor microenvironment and immune cell infiltration. Specifically, there was a positive association between the risk model and naïve B cells, resting dendritic cells and gamma delta T cells, while it was negatively related to CD8^+^ T cells. Finally, in vitro experiments, we found that UNC5B-AS1 inhibited OS cells from undergoing cellular senescence and apoptosis, thereby promoting OS cells proliferation. In conclusion, we constructed and verified a 5 AASR-based signature, that exhibited excellent performance in evaluating the overall survival of OS patients. In addition, we found that UNC5B-AS1 might inhibit the senescence process, thus leading to the development and progression of OS. Our findings may provide novel insights into the treatment of OS patients.

## Introduction

Comprising almost 60% of the frequent histological bone sarcoma subtypes, osteosarcoma (OS) is the most prevalent nonhematological malignancy that primarily influences children and adolescents^1,2^. OS is described as a cancer syndrome with a differentiation defect that originates from interstitial cells^3,4^. Multiple pieces of literature concerning the underlying mechanism of OS have revealed the landscape of associated driver mutations, including genomic alterations of p53, p53 inactivation, and RB deletion^5^. Approximately 15–20% of OS patients have clinically detectable metastasis at presentation, with the lungs accounting for the majority of distant metastases^6^. Despite advances in OS management and treatment with the development of aggressive surgery and multiagent chemotherapy, the prognostic outcomes have not improved as much as those of other malignancies over the past several decades^7^. As a consequence, OS remains the second leading cause of cancer-related death in children and adolescents^8^.

Cellular senescence is broadly described as an irreversible form of proliferative arrest that possibly evolves as a protective mechanism to inhibit the uncontrolled proliferation of tumor cells^9^. It has long been viewed as the main contributor to aging and aging-associated disorders^10^. Compared to young cells, such senescent cells display substantial morphological changes and decreased migratory abilities, which limit tumor invasion and metastasis^11^. Depending on the context, a dichotomy exists in the relationship between senescent cells and cancer, making the effects of senescent cells on cancer extremely complex. Among them, aging-associated genes (AAGs) play pivotal roles during the progression of cellular senescence, thus affecting tumor cells. On the one hand, they elicit tumor regression; on the other hand, they also exhibit permissiveness to infiltrative growth and other malignant behaviors of tumors^12,13^. In recent years, the oncology field has witnessed a burgeoning literature regarding the adoption of AAGs as clinical molecular biomarkers^14^.

As a subclassification of noncoding RNAs (ncRNAs), long ncRNAs (lncRNAs) are highly heterogeneous RNA transcripts with a length > 200 nucleotides^15^. An increasing number of studies indicate that lncRNAs play integral roles in the occurrence and development of diverse tumors^16,17^. Additionally, lncRNAs are involved in mechanisms linked with aging including proliferation, differentiation, and apoptosis^18^. Recent studies have revealed that lncRNAs are directly linked to senescence. The lncRNA MIR31HG has a dual role in senescence by suppressing CDKN2A expression in young cells and facilitating the production of a distinct subset of senescence-associated secretory phenotype (SASP) factors in senescent cells^19^. LncEPAT was reported to be a functional oncogene in glioblastoma, and lncEPAT silencing increased the expression of cell aging-associated genes, such as CDKN1A, CLUSTERIN, and DKK1, indicating that lncEPAT exerts a repressive function in glioblastoma cell senescence^20^. In addition, overexpression of lncRNA PANDA promoted hepatocellular carcinoma proliferation and carcinogenesis^21^. Mechanistically, PANDA impaired the transcriptional activity of senescence-associated inflammatory factor IL8, which led to the inhibition of cellular senescence. Recently, Zhang et al. performed a comprehensive bioinformatic analysis on aging-associated lncRNA signatures for predicting prognosis and immune status in glioma^22^, which may contribute to individualized treatment for glioma patients. However, to date, no studies have focused on aging-associated lncRNA signatures and their roles in molecular mechanisms or prognosis prediction in OS.

In the present study, we screened aging-associated lncRNAs in OS and performed bioinformatics analysis to establish a prognostic model that is capable of accurately predicting OS survival. Moreover, we experimentally demonstrated that UNC5B-AS1 can inhibit the senescence process of OS cell lines.

## Methods and material

### Data collection

The transcriptome data, mutation data and corresponding clinicopathological information of OS patients were downloaded from the TCGA database (https://portal.gdc.cancer.gov/). Among them, patients without survival information were removed for further analysis. A total of 88 OS patients were finally included, and the internal information of these patients is shown in Supplementary Table 1.

### Aging-associated survival lncRNA acquisition

LncRNAs and mRNAs were annotated according to the Ensemble Genome Browser website, and then their expression matrix was extracted separately. A total of 307 human AAGs were obtained from the Human Aging Genomic Resources (HAGR) database (http://genomics.senescence.info/) to construct the AAG expression matrix of OS patients^23^. Then, the expression correlation between lncRNAs and AAGs was analyzed to collect the aging-associated lncRNAs. LncRNAs with *p* < 0.001 and |Pearson correlation coefficient|> 0.4 were defined as aging-associated lncRNAs. Subsequently, univariate Cox proportional hazards regression analysis and Kaplan‒Meier survival analysis were performed to identify (*p* < 0.001) aging-associated survival lncRNAs (AASRs).

### Consensus clustering analysis

Consensus clustering was performed on AASRs using ConsensusClusterPlus v1.38. Based on the consensus cumulative distribution function (CDF) and the delta area, two clusters were identified. Survival analysis was performed between the two clusters to estimate their impact on OS prognosis using the ‘survminer’ package. In addition, the expression patterns of the 10 AASRs in the two clusters were visualized in a box plot. Gene set variation analysis (GSVA) is a nonparametric and unsupervised enrichment method that is capable of evaluating the variation in biological process activity and pathways through transcriptomic data^24^. For the GSVA analysis, the ‘GSEABase’ and ‘GSVA’ packages were used to investigate the pathway enrichment between the two clusters.

### Construction of the lncRNA Signature

The 10 AASRs were used for least absolute shrinkage and selection operator (LASSO)-Cox regression analysis to construct the risk model with the ‘glmnet’ package. The aging-associated risk scores were calculated by the following formula: Risk score = β1^∗^X1 + β2^∗^X2 + $\cdots$ + βn^∗^Xn. β is the regression coefficient by LASSO-Cox regression, and X is the expression of core prognostic genes. Then, based on the median risk score, OS patients were stratified into high-risk (n = 44) and low-risk (n = 44) groups. Kaplan‒Meier analysis and a two-sided log-rank test were performed to determine the difference in overall survival between the high- and low-risk groups. In addition, receiver operating characteristic (ROC) curve analysis was used to assess overall survival for clinicopathological characteristics (including age, gender, and metastases) and risk scores.

### Evaluation of the lncRNA Signature

The concordance index (C-index) method was used to estimate the predictive ability of the risk scoring signature with the ‘rms’ and ‘survival’ packages. The clinical significance of the risk signature was evaluated among different clinical characteristics by decision curve analysis (DCA). OS samples were ranked based on risk scores, and dot-plots of survival status were drawn for each case. Moreover, the expression profiles of lncRNAs in the signature between the two groups were visualized with the ‘pheatmap’ package.

### Independence prognostic analysis

Univariate and multivariate Cox regression analyses were used to determine independent prognostic factors for OS patients. Next, based on these clinical factors (age, gender, metastasis) and risk score, a nomogram was established to assess the 1-, 3-, and 5-year overall survival of OS patients. In addition, survival analysis stratified by clinical subgroups between the high-risk and low-risk groups was performed.

### Comprehensive analysis

The ESTIMATE algorithm was implemented to appraise the proportion of immune/stromal components to identify the tumor microenvironment (TME) characteristics of OS samples^25^. The CIBERSORTx algorithm was used to quantify the proportion of immune cells in the OS samples. Then, the correlation between immune cell infiltration and risk scores was explored. To explore the sensitivity of chemotherapeutic agents between high- and low-risk groups, the ‘pRRophetic’ package was utilized. The sensitivity of chemotherapeutic agents is presented as the half maximal inhibitory concentration (IC50). Then, gene set enrichment analyses (GSEA) was performed using GSEA software, to explore the underlying biological processes of the model. The top five enrichment terms in the high- or low-risk groups are presented. Finally, the ceRNA networks of GAS5 and UNC5B-AS1 were successfully constructed through the ENCORI database (https://starbase.sysu.edu.cn/) and presented with Sankey diagrams. Moreover, we chose UNC5B-AS1 for functional analyses.

### Cell culture and knockdown of UNC5B-AS1

The human OS cell line U2OS was purchased from the Chinese Academy of Sciences (Shanghai, China) and cultured in McCoy’s 5a medium (Thermo Fisher, USA) with 10% fetal bovine serum (HyClone, USA), 100 units/ml penicillin, and 100 μg/ml streptomycin in a 5% CO_2_ atmosphere at 37 °C. Small interfering RNA (siRNA) targeting UNC5B-AS1 was transfected using Lipofectamine 3000 (Life Technologies, USA) at a final concentration of 20 nM according to the manufacturer’s instructions. The siRNA sequences of UNC5B-AS1 siRNA (5′-GGGAAGUGCCCUUACCCUATT-3′) and control siRNA (5′-UUCUCCGAACGUGUCACGUTT-3′) were constructed by Gene Pharm Company (Shanghai, China).

### RNA extraction and quantitative real-time PCR

Quantitative real-time PCR was performed to test UNC5B-AS1 expression in U2OS cells. TRIzol reagent (Invitrogen Life Technologies) was used to extract total RNA from U2OS cells according to the manufacturer’s protocol. A NanoDrop 2000 (Thermo Scientific) was utilized to test the concentration and purity of total RNA. First, the PrimeScript RT Reagent Kit (TaKaRa) was used to synthesize cDNA from 1 μg of total RNA from the samples. SYBR Premix Ex Taq II (TaKaRa) was used to carry out quantitative real-time PCR in a QuantStudio™ 3 System. The process of PCR was performed using the following conditions: 40 cycles at 95 °C/30 s, 95 °C/15 s, and 60 °C/30 s. The data were finally analyzed by 2^−ΔΔCt^. The primers were as follows: UNC5B-AS1 (F: 5′-GATGCTAATCAGGCCGCTAAGATGG-3′; R: 5′-CCCACCCCTTTTGTTTCCTCTTCC-3′), GAPDH (F: 5′- CAGGAGGCATTGCTGATGAT-3′; R: 5′-GAAGGCTGGGGCTCATTT-3′).

### Protein isolation and western blotting analysis

Total proteins were extracted by RIPA lysis buffer containing protease inhibitor mix (1 mM PMSF, 2 μg/ml Roche protease inhibitor cocktail) . A BCA kit (Biyuntian, China) was used to detect the protein concentration. The following primary antibodies were used: anti-P21 (Abcam, ab109520), anti-PCNA (Abcam, ab29) and β-tubulin (Abcam, ab6046) as controls. The primary antibodies were incubated with PVDF membranes overnight at 4 °C according to the manufacturers' recommendations. The membrane was cut according to the respective protein molecular weight before incubation of the primary antibody. After that, the secondary antibodies were incubated with the membranes, and imagining analysis was performed.

### SA-β-gal staining and EdU assay

SA-β-gal staining was performed to determine the level of SA-β-gal expression in U2OS cells using a kit purchased from Sigma‒Aldrich (Germany) according to the manufacturer's instructions. Briefly, U2OS cells were harvested and fixed with fixation buffer, washed three times in PBS and permeabilized prior to staining. After that, the cells were stained with SA-β-gal staining solution overnight at 37 °C. Finally, SA‐β‐gal‐positive cells, stained blue, were randomly imaged. For cell proliferation analysis, EdU detection kit (RiboBio, C10310) was used to measure the difference in live cell count and cell percentages between different groups. An overnight incubation with EdU reagent solution at 37 °C followed by 10 min of fixation with 4% paraformaldehyde was carried out in U2OS cells. The following day, the cells were subjected to imaging analysis and cell counting. For statistical analysis, this formula calculated the percentage of EdU-positive cells based on the count of EdU-positive cells (EdU-positive cells + EdUu-negative cells).

### Flow cytometry

The effect of UNC5B-AS1 on OS cell apoptosis was determined with an annexin V-APC apoptosis detection kit (Procell Life Science & Technology Co.,Ltd, cat: P-CA-202), and the analysis was performed according to the manufacturer's instructions. U2OS cells were washed twice using PBS solution. Cells were then resuspended in binding buffer and adjusted to a concentration of 1 × 10^6^. After adding 5 μL of Annexin V-APC and incubating on ice for 10 min, 10 μL of 7-AAD was added and incubated on ice for 5 min in the dark. The incubated cells were washed and resuspended in binding buffer and then tested using FACS. The results were analyzed using BD software.

### Statistical analysis

Statistical analysis in the current study was performed using R (v.3.8.0). The data are presented as the mean values ± standard. Student’s two-tailed t test was used to calculate statistically significant differences between the two groups. One-way ANOVA was used to analyze the differences between more than three groups. P values less than 0.05 were used as cutoff criteria.

### Ethical approval

All methods were carried out in accordance with relevant guidelines and regulations.

## Results

### Identification of AASRs

**Figure 1:**
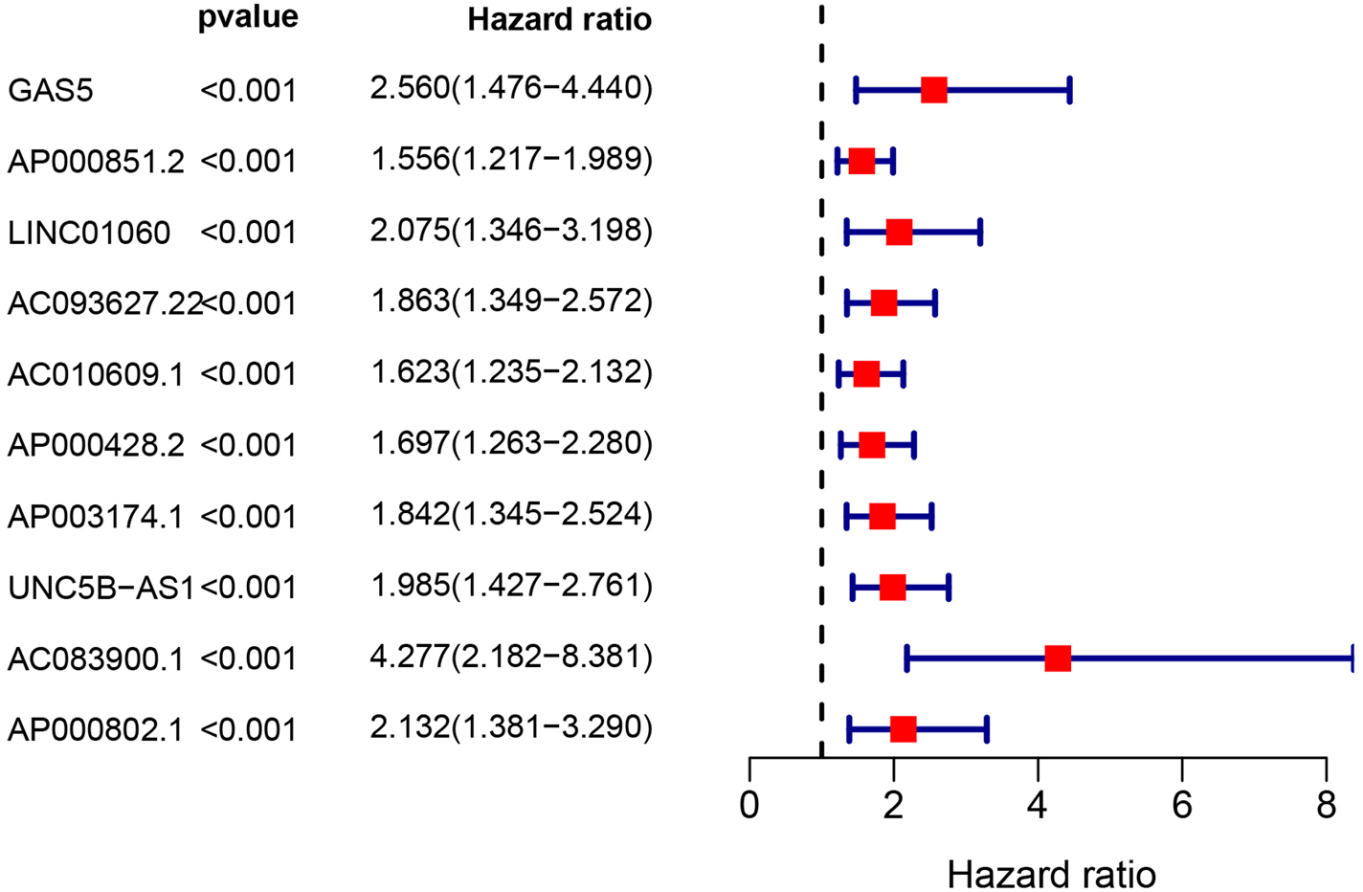
Forest plots of the 10 AASRs in OS samples.

To determine AASRs, we first obtained 307 AAGs from the HAGR database. We then calculated the AAG expression matrix in 88 OS samples and investigated the correlations between lncRNAs and AAGs with Pearson correlation analysis, thereby acquiring 1236 aging-associated lncRNAs. Next, univariate Cox regression analysis was performed and only 10 aging-associated survival lncRNAs (namely 10 AASRs, including GAS5, AP000851.2, LINC01060, AC093627.22, AC010609.1, AP000428.2, AP003174.1, UNC5B-AS1, AC083900.1 and AP000802.1), which acted as risk factors (HR > 1), were found to be significantly associated with the overall survival of OS patients (*p* < 0.001, Fig. 1).

### Consensus clustering analysis

**Figure 2:**
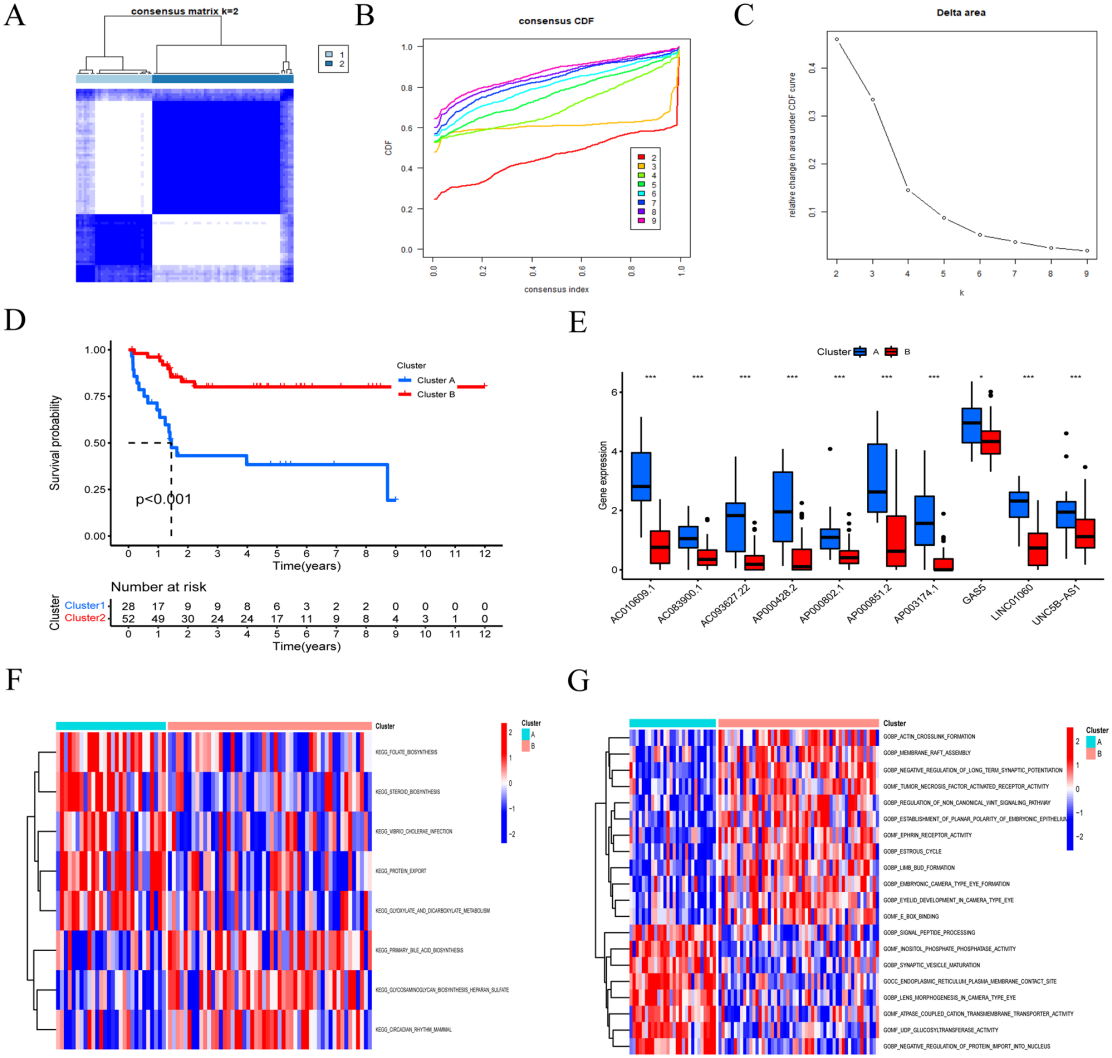
Consensus clustering analysis. (**A**) Consensus score matrix of all samples when k = 2. (**B**) The cumulative distribution functions (CDF) for k = 2–9. (**C**) Relative change in the area under the CDF area for k = 2–9. (**D**) Survival analysis of the two clusters. (**E**) Box plot showing the expression levels of 10 AASRs between the two clusters. (**F** and **G**) GSVA analysis by KEGG and GO enrichment pathway analysis in two clusters.

Based on the expression levels of the 10 AASRs, the OS patients were classified into two clusters (C1: Cluster A, C2: Cluster B) with consensus clustering analysis (Fig. 2A), with the consensus matrix for optimal k = 2 (Fig. 2B, C). Then, survival analysis showed that Cluster A had a worse prognosis, while Cluster B had a better prognosis (Fig. 2D). Moreover, the box plot shows the expression levels of the 10 AASRs, and all these AASRs were significantly upregulated in Cluster A (Fig. 2E), indicating possible crucial roles in OS progression and prognosis.

Here, to push our investigations further, we sought to decode the underlying molecular mechanisms leading to differential prognosis for OS in two clusters by performing GSVA analysis. AASRs in Cluster B were largely abundant in GO terms of actin crosslink formation, membrane raft assembly, negative regulation of long-term synaptic potentiation, tumor necrosis factor activated receptor activity, regulation of noncanonical Wnt signaling pathway, and so on (Fig. 2G). On the other hand, AASRs in Cluster A were mostly enriched in signal peptide processing, inositol phosphate phosphatase activity, synaptic vesicle maturation, and so on (Fig. 2G). The results of KEGG analysis showed that AASRs in Cluster A were mainly enriched in folate/steroid biosynthesis, vibrio cholerae infection, protein export, and glyoxylate and dicarboxylate metabolism pathways (Fig. 2F). AASRs in Cluster B were typically enriched in primary bile acid biosynthesis, glycosaminoglycan biosynthesis heparan sulfate, and circadian rhythm mammal pathways (Fig. 2F).

### Construction of the lncRNA signature

**Figure 3:**
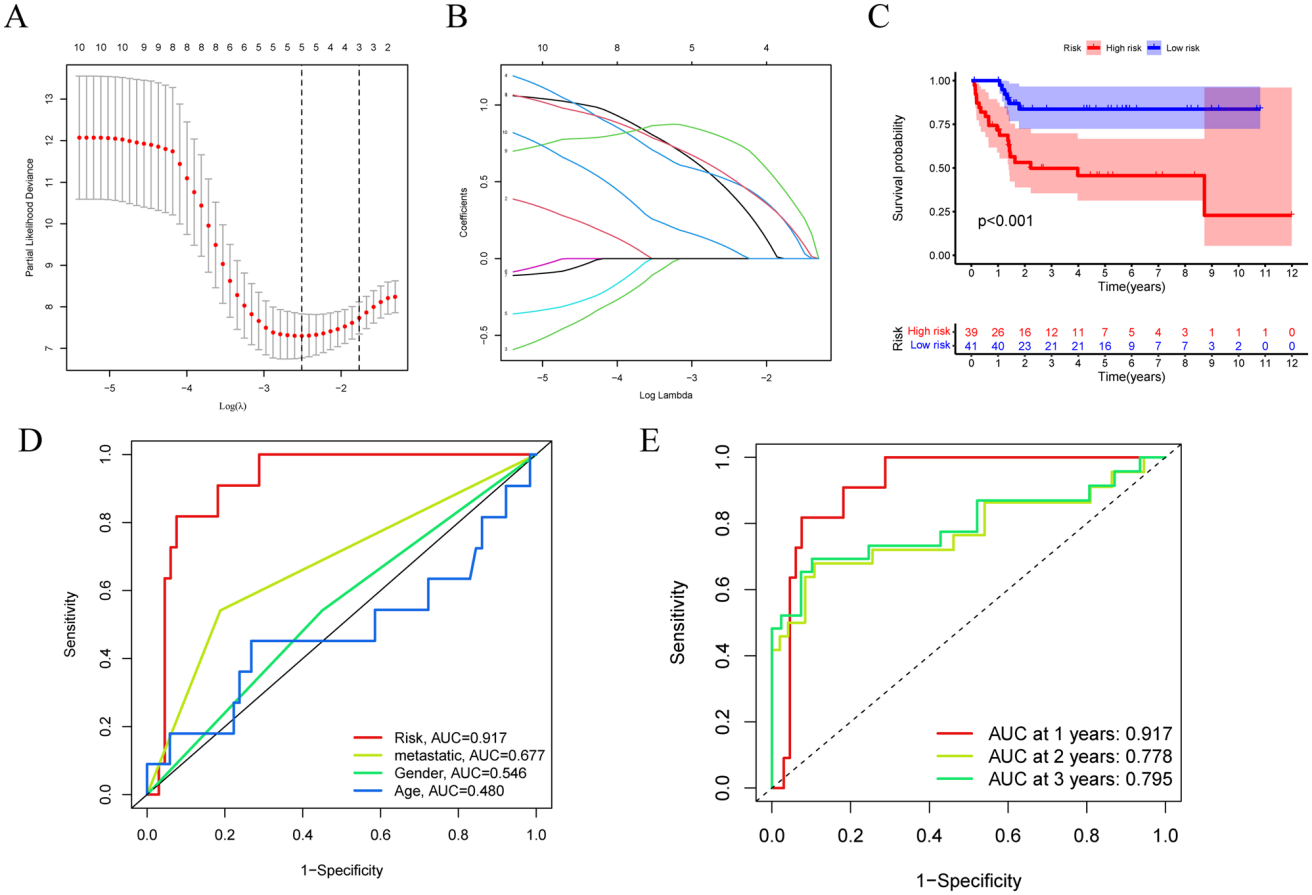
Construction of the lncRNA signature. (**A**) The LASSO coefficient of the 10 lncRNAs in OS. (**B**) Select the best parameters for OS on the basis of LASSO. (**C**) Kaplan‒Meier survival curves of the overall survival of OS patients in the high-risk and low-risk groups. (**D**) ROC curve of clinicopathological features, including age, gender, metastasis, and riskScore. (**E**) ROC curve of the risk signature for predicting 1-year, 2-year, and 3-year survival.

The 10 AASRs were subjected to LASSO analysis to generate an aging-associated lncRNA signature, and 5 AASRs were ultimately included in the signature (Fig. 3A and B). Then, the patients were divided into high-risk and low-risk groups. Kaplan‒Meier survival analysis verified that the low-risk group exhibited a more favorable prognosis than the high-risk group (Fig. 3C). Indeed, numerous studies have reported that clinical characteristics (e.g., age, gender, and metastasis) are involved in the progression and prognostic development of OS patients^26,27^. Having performed a time-dependent ROC analysis among the clinical factors (including age, gender, metastasis, and riskScore), we found that the riskScore had excellent accuracy in predicting the survival of OS patients, with an area under the curve (AUC) of 0.917 (Fig. 3D). Furthermore, the model had the best accuracy in 1-year survival prediction (Fig. 3E).

### Evaluation of the lncRNA signature

**Figure 4:**
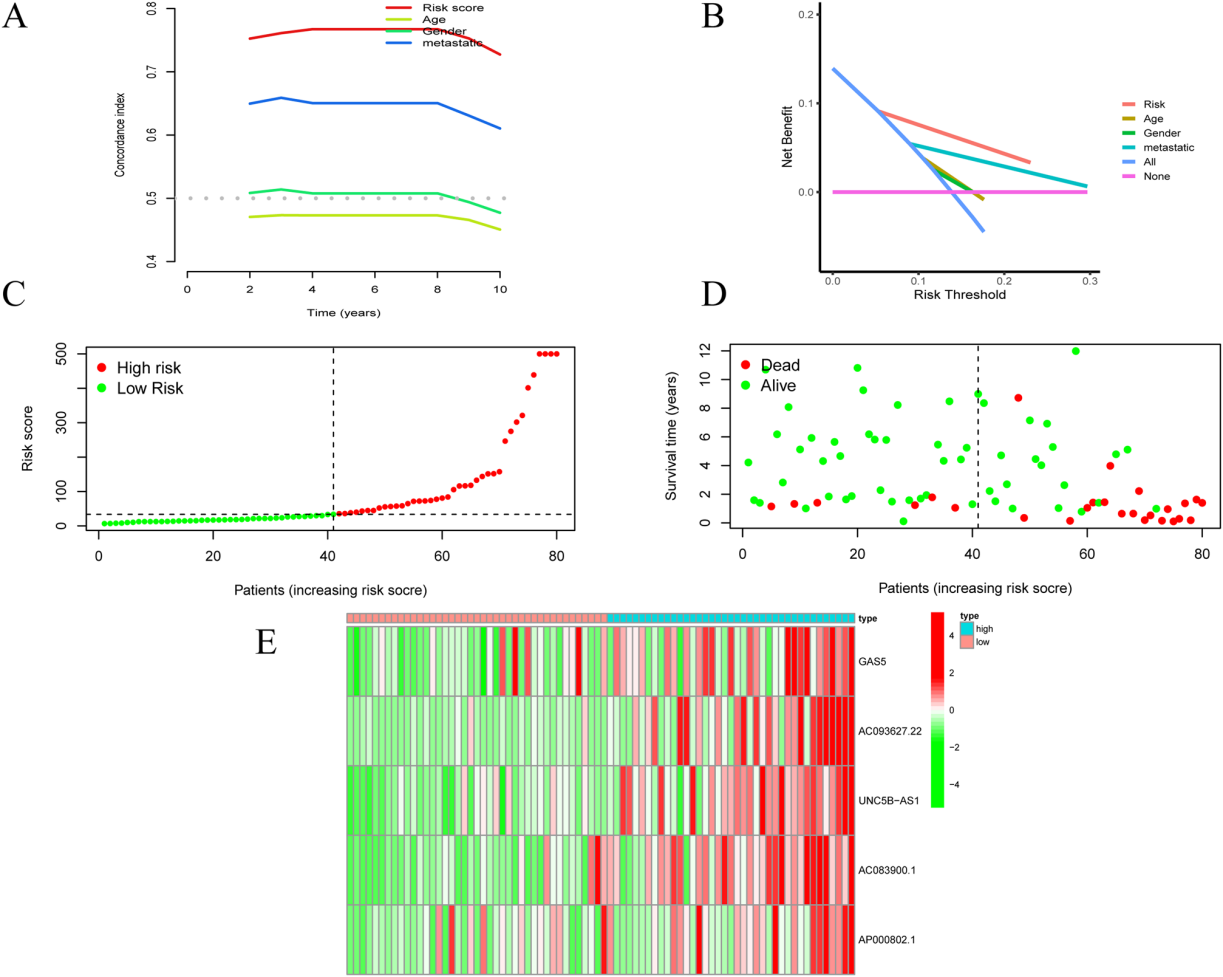
Evaluation of the risk model. (**A**) C-index and (**B**) DCA analysis. (**C**) The survival time and (**D**) survival status of OS patients in the high-risk and low-risk groups. (**E**) Heatmap of the expression patterns of the 5 AASRs in the high-risk and low-risk groups.

To further measure the efficacy of the predictive probability of the risk signature, we performed C-index and DCA analyses (Fig. 4A and B). The results showed that the riskScore had the best prediction accuracy and efficiency among all these factors (riskScore, age, gender, metastasis). We also found that the riskScore could well distinguish the overall survival of OS patients. According to the AASRs signature, the risk score distribution and survival status of each OS sample in the high-risk group and the low-risk group were visualized (Fig. 4C, D). The results implied that the higher the risk score of patients, the higher the mortality rate and the shorter the survival time. In addition, the heatmap showed the expression patterns of the 5 AASRs (GAS5, AC093627.22, UNC5B-AS1, AC083900.1, and AP000802.1) in the model between the high-risk and the low-risk groups, and all 5 AASRs were upregulated in the high-risk group (Fig. 4E).

### Identification of independent prognostic factors

**Figure 5:**
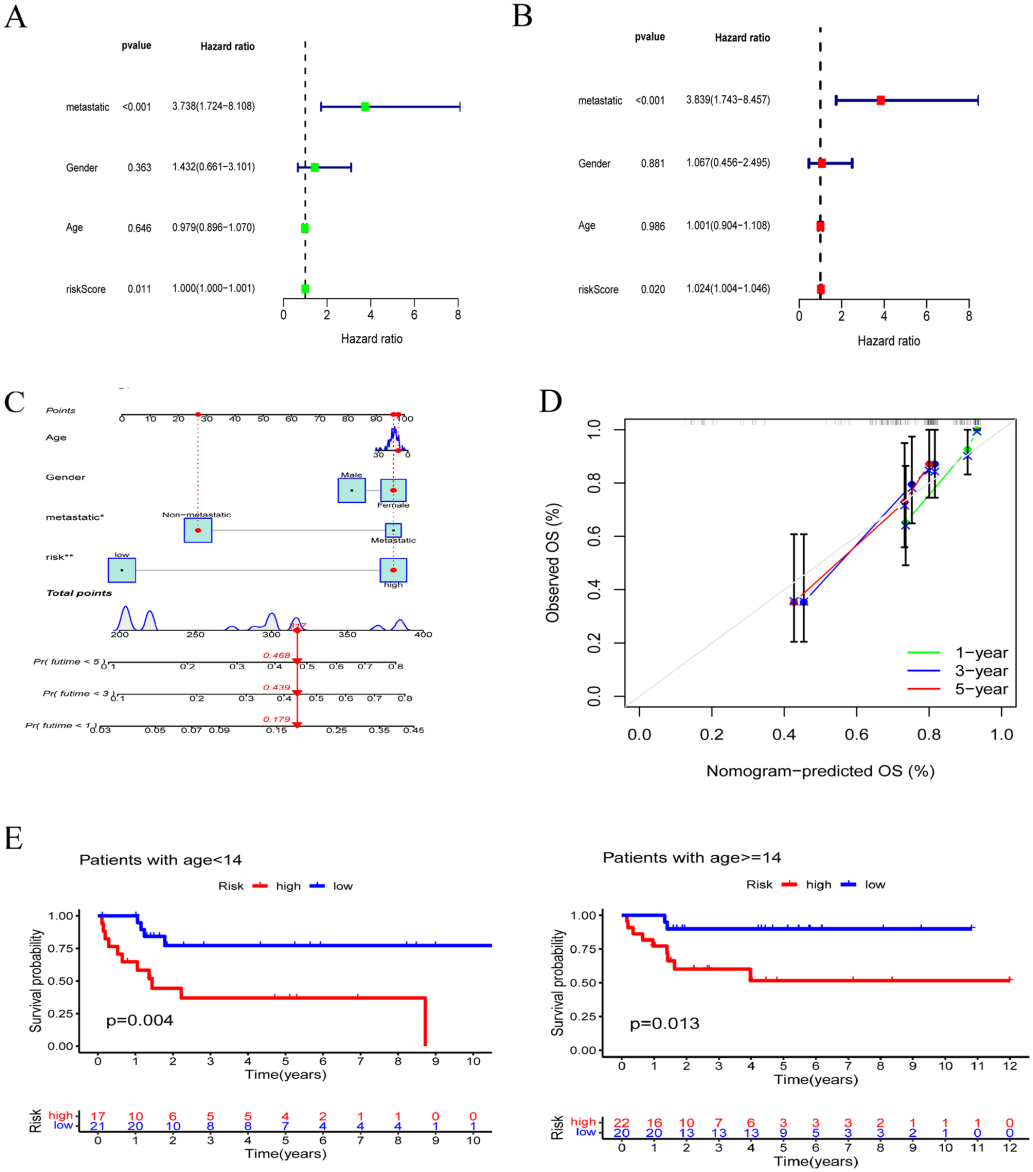

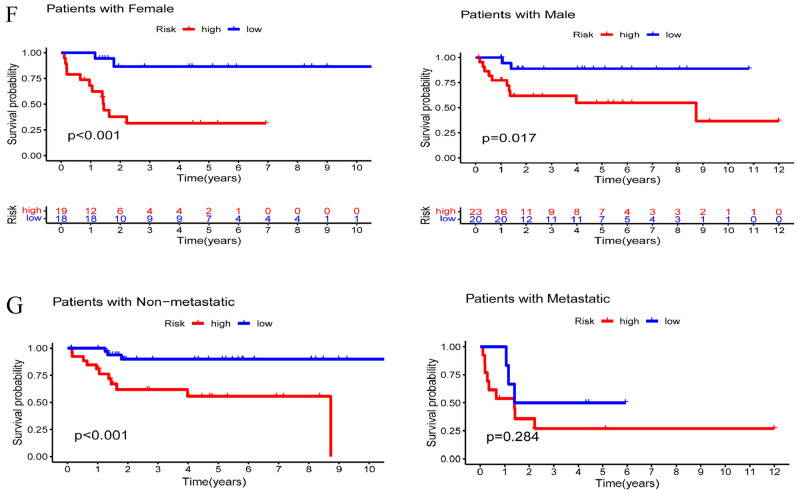
Identification of independent prognostic factors and stratified survival by clinical subgroups. (**A**) Univariate and (**B**) multivariate Cox regression analysis to identify independent prognostic factors. (**C**) A nomogram was constructed based on the clinical factors and riskScore. (**D**) Calibration curve of the nomogram for 1/3/5-year overall survival probabilities of patients with OS. (**E**–**G**) Survival analysis for the high-risk and low-risk groups by clinical factors (age, gender and metastasis).

By carrying out univariate and multivariate Cox regression analyses, we observed that metastasis was a prominent independent prognostic factor for OS patients (all *P* < 0.001) (Fig. 5A, B). In addition, the riskScore also acted as an independent prognostic factor, although the P values were not as significant as those for metastasis (*P* = 0.011 and 0.020, respectively). Furthermore, we also developed a nomogram that can be used to assist clinicians in making decisions about patients with OS. Age, gender, and metastasis were included in the nomogram along with the risk scores. Among these factors, the risk model had the predominant predictive ability in the nomogram (Fig. 5C). Alternatively, we mapped a calibration curve to evaluate the 1/3/5-year survival rates, and the risk model exhibited encouraging performance in predicting OS survival (Figs. 5D).

We also performed survival analysis for the high-risk and low-risk groups by clinical factors (age, gender and metastasis). The results revealed that high-risk OS patients had worse survival times than low-risk patients in the age (age < 14, age ≥ 14) and gender (male, female) subgroups, indicating that the risk model had good predictive performance in OS patients in these two different subgroups, regardless of the patient's age and gender (Fig. 5E, F). In nonmetastasis subgroup, patients with a high risk score had a worse survival probability, while the metastasis subgroup did not have such a peculiarity, which may be due to the fewer metastasis samples (only 19 cases) (Fig. 5G). This result may be further verified when more cases are available.

### TME and immune infiltration analysis

**Figure 6:**
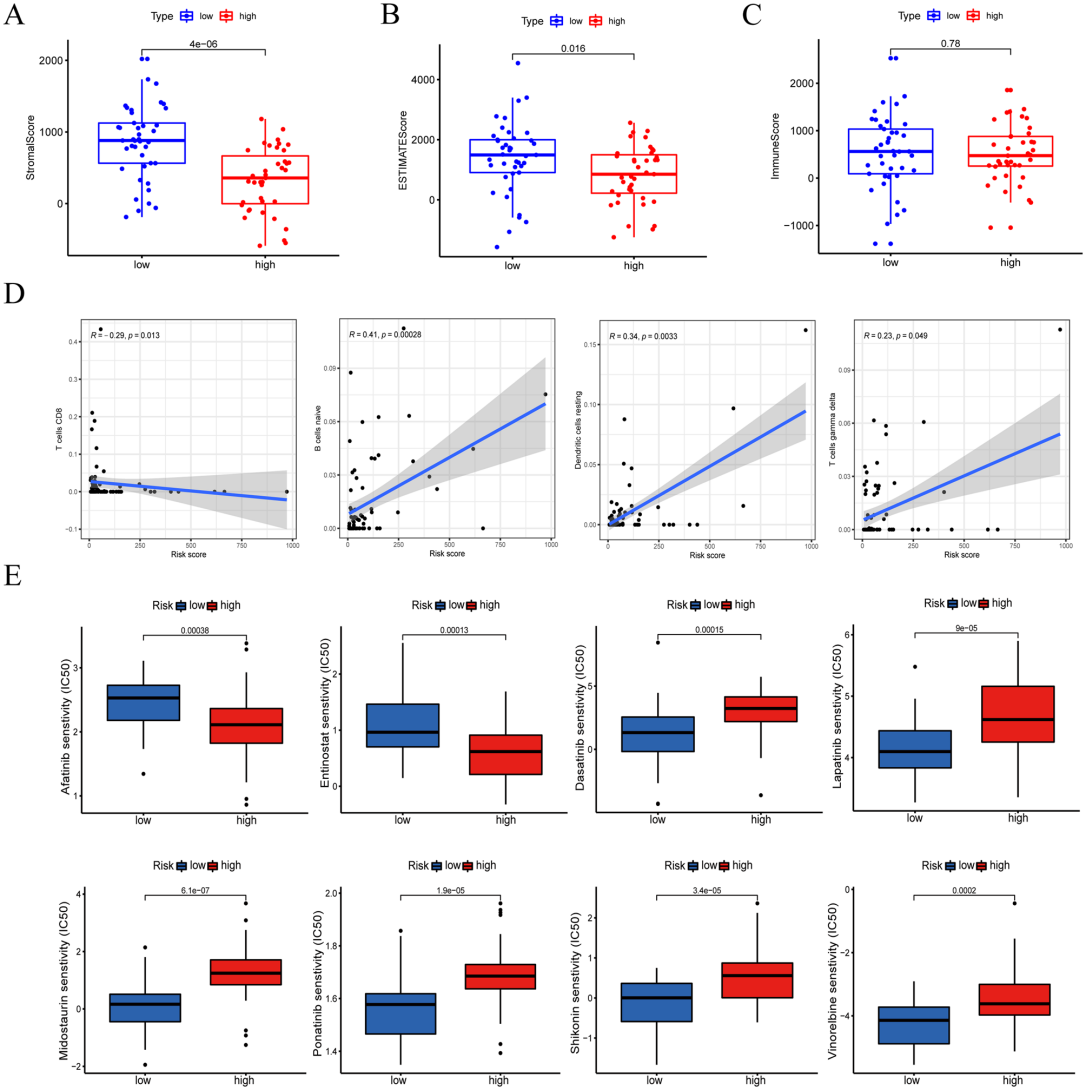
TME characteristics and immune infiltration analysis. (**A**) Stromal score, (**B**) ESTIMATE score, and (**C**) immune score between the high-risk and low-risk groups. (**D**) The risk score was positively associated with naïve B cells, resting dendritic cells and gamma delta T cells but negatively related to CD8^+^ T cells. (**E**) Drug sensitivity analyses between the high-risk and low-risk groups.

An increasing number of studies have suggested that the TME, shaped by tumor cells that are permissive for their survival and growth, plays an integral role during the neoplasia of OS^28,29^. Thus, to illustrate the TME characteristics and immune landscape of OS, we carried out a series of analyses. With the ESTIMATE algorithm, the immune, stromal and ESTIMATE scores for the OS samples were calculated. The stromal scores and ESTIMATE scores were significantly higher in the low-risk group than in high-risk group (*P* < 0.05) (Fig. 6A–B). Likewise, the immune score difference between the two groups showed a similar tendency, although there was no statistical significance (*P* > 0.05) (Fig. 6C). Additionally, we investigated the relationship between the risk score and immune cells. The risk score was positively related to naïve B cells, resting dendritic cells and gamma delta T cells but negatively correlated with CD8^+^ T cells (Fig. 6D).

As drug sensitivity can also affect outcomes during OS chemotherapy, sensitivity analyses were conducted to unveil the treatment effects between the high-risk and low-risk groups. We found that patients in the high-risk group exhibited higher sensitivity to afatinib and entinostat than the samples in the low-risk group but showed higher sensitivity to dasatinib, lapatinib, midostaurin, ponatinib, shikonin and vinorelbine (Fig. 6E).

### GSEA and construction of the ceRNA network

**Figure 7:**
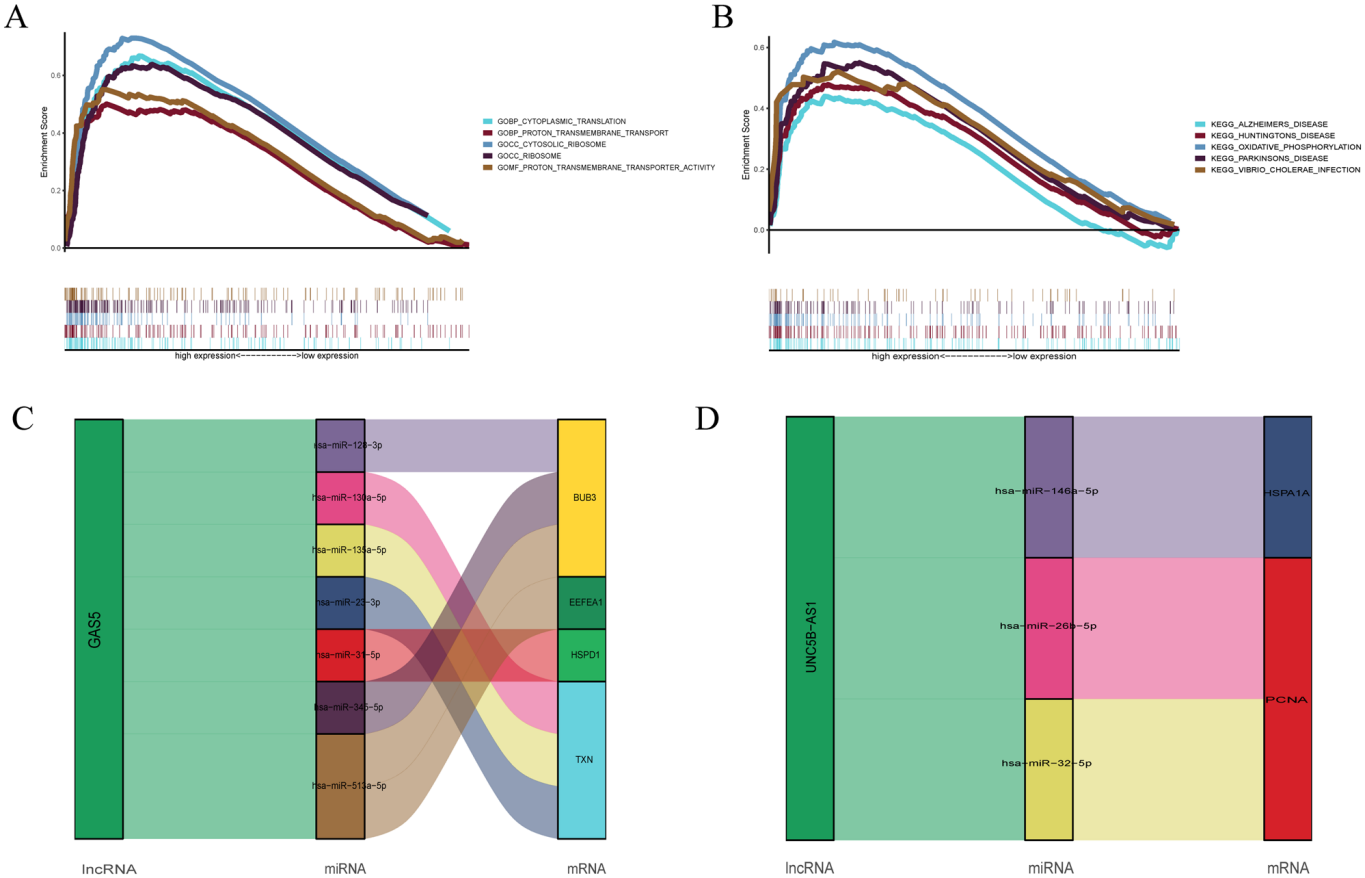
GSEA and ceRNA network establishment. (**A**) Significantly enriched GO terms of the risk model. (**B**) Significantly enriched KEGG pathways of the risk model. (**C**) CeRNA network construction based on GAS5. (**D**) CeRNA network construction based on UNC5B-AS1.

We then performed GSEA to explore the underlying biological processes of the risk model. Intriguingly, the model was significantly enriched in GO terms of cytoplasmic translation, while KEGG pathways principally centered on Alzheimer's disease (Fig. 7A and B). Then, among the 5 AARs, only the ceRNA networks of GAS5 and UNC5B-AS1 were successfully established to show the interaction between the lncRNAs, miRNAs and mRNAs. Currently, GAS5 is becoming an increasingly interesting lncRNA in cancer research and management^30^. As shown in Fig. 7C and D, more miRNAs connected to GAS5 than the other lncRNA UNC5B-AS1, indicating that GAS5 might have an important role in OS though the ceRNA network. For UNC5B-AS1, the ceRNA network showed that it could target miR-32-5p or miR-26b-5p to regulate the expression of PNCA. Finally, we chose UNC5B-AS1 for further study because its biological function has rarely been reported in OS.

### Functions of UNC5B-AS1 in OS progression

**Figure 8:**
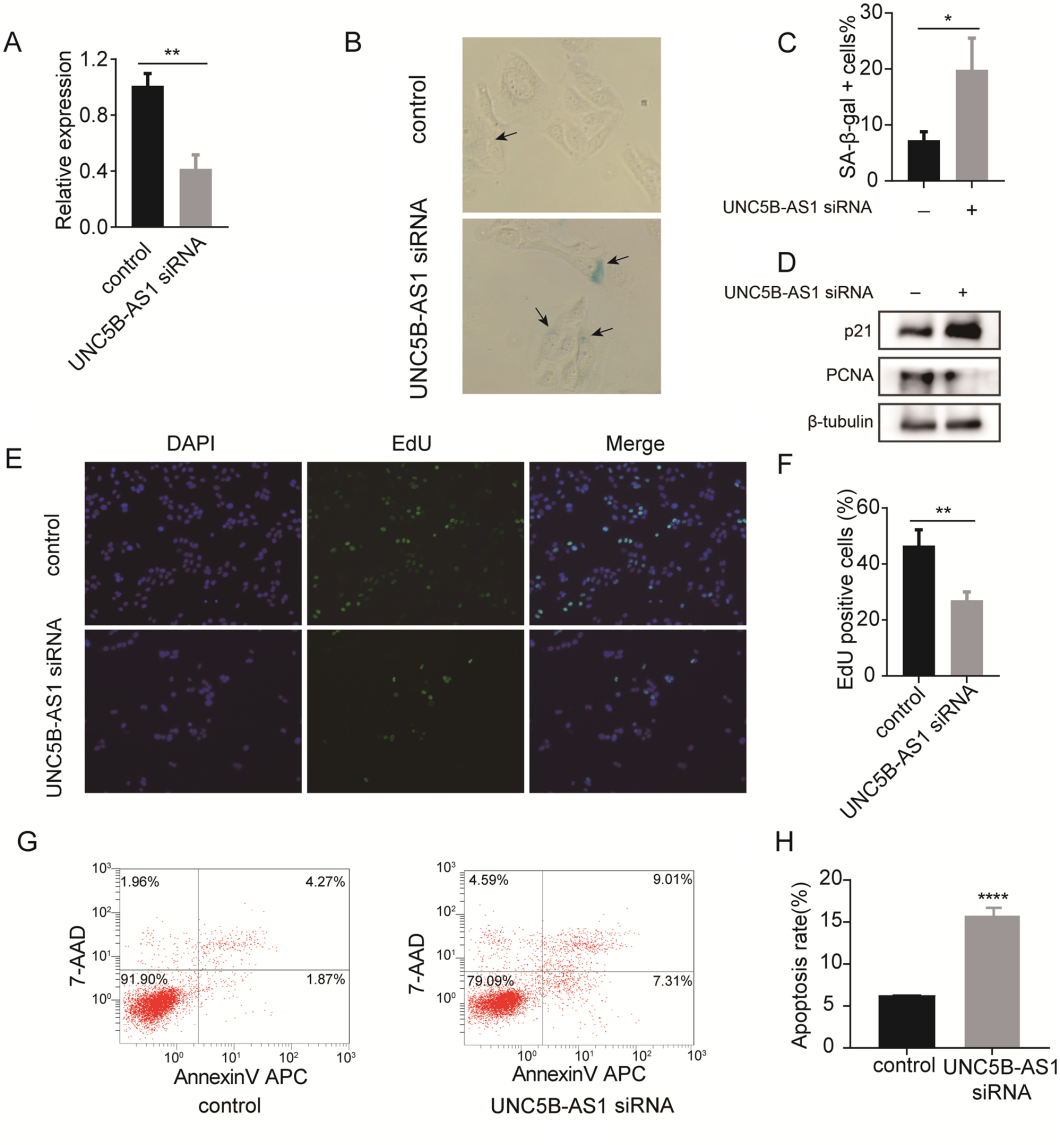
UNC5B-AS1 knockdown inhibited OS cell proliferation and promoted cellular senescence and apoptosis. (**A**) qPCR showed the transfection efficiency of UNC5B-AS1 siRNA. (**B**, **C**) SA-β-gal staining in UNC5B-AS1 siRNA and control groups. (**D**) Western blot showing the protein expression of p21 and PCNA (cut before incubation of the primary antibody). (**E**, **F**) EdU assay showing the proliferation capacity of OS cells. (**G**, **H**) Flow cytometry revealed the apoptosis of U2OS cells.

U2OS cells were transfected with UNC5B-AS1 siRNA for further loss-of-function experiments and the transfection efficiency was determined via qPCR (Fig. 8A). SA-β-gal activity was measured using SA-β-gal staining and the degree of cellular senescence was assessed by the SA-β-gal-positive ratio. The results showed that cellular senescence was significantly increased in UNC5B-AS1 knockdown cells (Fig. 8B and C). When UNC5B-AS1 was knocked down, the expression of p21 protein was significantly enhanced, while the expression of PCNA protein was dramatically decreased (Fig. 8D) (Supplementary Fig. 1). This means that UNC5B-AS1 knockdown could promote cellular senescence and inhibit the growth in U2OS cells. Then, to further confirm this, the EdU assay also showed that downregulation of UNC5B-AS1 inhibited OS cell proliferation (Fig. 8E and F). Finally, flow cytometry revealed that downregulating UNC5B-AS1 significantly increased apoptosis of the U2OS cells (Fig. 8G, H). Taken together, UNC5B-AS1 might inhibit cellular senescence and apoptosis, thereby promoting cell proliferation.

## Discussion

Conventional clinical analysis has limitations in that it fails to accurately and dynamically reflect OS progression and predict unfavorable prognostic capacity. Consequently, adequate and precise estimation and prediction of biomarkers that capture disease progression are indispensable for therapy decision-making and survival evaluation in OS patients. Currently, the correlation between aging and OS remains unclear. In general, genomic mutations and, in particular, DNA damage as a result of abnormal proliferation, accumulate with age^31^. Additionally, instigators such as hyperactivated signaling-induced, chemotherapy-induced, and radiotherapy-induced senescence contribute to malignancy progression^32^. Among the biological functions and structures that are progressively affected by aging, bone is of great significance, given its fundamental role as a crucial endocrine and immune organ^33^. The dysfunction of bone caused by OS is orchestrated by the TME. Aging is strongly correlated with abnormal immunity and an accumulated chronic inflammatory microenvironment, thus facilitating OS development and progression.

Multiple lines of evidence have established the critical role of aging during the development and progression of various tumors. It is worth noting that lncRNAs are pivotal regulators during aging^34,35^. Moreover, lncRNAs have been increasingly appreciated for their potential as prognostic candidates for OS^36^. In the present study, we acquired 1236 aging-associated lncRNAs, and a total of 10 AASRs were ultimately discriminated with univariate Cox regression analysis. Having performed cluster analysis, we explored the difference in survival outcomes between the two clusters. It was apparent that Cluster B presented a better prognosis. The pathway enrichment analysis showed different molecular signaling patterns between the two clusters. Furthermore, 5 AASRs (GAS5, AC093627.22, UNC5B-AS1, AC083900.1, and AP000802.1) were identified by LASSO regression and used to construct a risk model to predict the overall survival of OS. Interestingly, the risk model had good predictive performance in predicting OS prognosis. The C-index and DCA analysis further confirmed the superior prediction accuracy and efficiency of the model. The higher the risk score, the the higher the mortality rate and the shorter the survival time. Subsequently, the riskScore was identified as an independent factor, and the nomogram demonstrated the predominant ability to predict OS survival. Additionally, survival analysis for age and gender subgroups showed that the high-risk OS patients had adverse survival times compared with the low-risk group.

The TME consists of various types of cells mediating the communication between malignant cells, immune cells and stromal cells^37^ and provides all metabolites and factors for controlling proliferation, dissemination, dormancy, and drug resistance in OS cells^38^. The ESTIMATE algorithm showed that stromal scores and ESTIMATE scores were significantly higher in the low-risk group than in the high-risk group, while the immune score difference showed a similar tendency without statistical significance. It has been discovered that tumor-infiltrating immune cells are significantly relevant to the progression and prognosis of OS^39^. Our study further suggested that the risk score was positively related to naïve B cells, resting dendritic cells and gamma delta T cells but negatively correlated with CD8^+^ T cells. B cells, as precursors of antibody-producing plasma cells, could interact with other immune cells through cytokine secretion and antigen presentation. The specific signals drive the differentiation of human naive B cells into memory B cells and plasma cells, all of which play distinct roles during humoral immune responses^40^. Most studies have reported that the presence of B cells, assessed using either genomics or cellular approaches, has been associated with an improved outcome in cancer patients, and impaired antitumor immunity, predominantly through the generation of immunosuppressive cytokines^41^. Dendritic cells bridge the gap between innate and adaptive immunity and maintian immune responses against malignant cells. A recent study reported that high levels of resting dendritic cells indicated lower levels of immunoreaction, while activated dendritic cells may contribute to improving OS survival rate^42^. Previous studies have identified gamma delta T cell as the prognostically most favorable immune cell subset in tumor infiltrates from 18,000 tumors across 39 malignancies^43^, and high gamma delta T-cell frequency in tumor infiltrates from cancer patients correlates with better clinical outcome in different malignancies^44,45^. CD8^+ ^ T cell infiltration not only inhibits growth and lung metastasis of OS, but is also significantly associated with favorable survival^46,47^. In this context, the risk model may play important roles in the TME of OS, thus affecting the prognosis of OS patients.

In addition, several chemotherapy drugs were proven to be sensitive to OS patients, suggesting the potential of our prognostic risk model in guiding multiagent chemotherapy. The results of the GSEA showed that the signature was mostly enriched in Alzheimer's disease, which is known as an aging-associated disease. A recent study found that the neurovascular unit in Alzheimer's disease had unique aging-associated transcriptomic profiles and cellular pathways compared with negative control brain cells^48^. Finally, the ceRNA networks of GAS5 and UNC5B-AS1 were sucessfully constructed. Of note, we chose UNC5B-AS1 for further study because its biological function has rarely been reported in OS. Indeed, GAS5 has been widely investigated in a variety of reports on tumors, including OS^49^. It is generally recognized as a tumor suppressor that is frequently downregulated in tumorigenesis, particularly in metastatic cancers^50,51^. An isolated study reported that GAS5 exerted its tumor suppressor function by interacting with the tumor suppressor genes p53 and Bax^52^. On the other hand, there is an interesting revelation that GAS5 was able to transduce miR-23a-3p to modulate OS progression, suggesting an interactive relationship between GAS5 and miRNAs, which was also consistent with our findings^53^. UNC5B-AS1 is a novel lncRNA related to the malignant progression of prostate cancer and can directly target downstream caspase-9, which is a key molecule in the tumor pathway^54^. Furthermore, there was a negative relationship between UNC5B-AS1 and caspase-9. In our study, UNC5B-AS1 was identified as a candidate AASR during OS progression. OS cell lines (U2OS) with knockdown of UNC5B-AS1 were constructed by cell transfections to investigate the function of UNC5B-AS1 in OS senescence and progression. SA-β-gal staining assays indicated that low expression of UNC5B-AS1 could promote cellular senescence in OS. The downregulation of UNC5B-AS1 was associated with overexpressed P21 and low-expressed PCNA. P21, as a crucial metabolic signal, activates PAKs to manipulate cell aging. For example, PAK-specific deficiency in a mouse model increased the survival time of mice and impaired age-related phenotypes^55^. In addition, P21 has been confirmed to trigger fibroblasts aging^55^. PCNA was discovered to serve a critical purpose in controlling the cell cycle, implying that the PCNA index was a marker of cell proliferation^56^. Above results indicated that knockdown of UNC5B-AS1 promoted cellular senescence and inhibited cell proliferation. The EdU assay also confirmed that downregulation of UNC5B-AS1 inhibited OS cell proliferation. It means that UNC5B-AS1 silencing may trigger cellular senescence, and then lead to the proliferation cessation. In addition, flow cytometry revealed that downregulating UNC5B-AS1 significantly increased apoptosis of U2OS cells, which may be caused by senescence. For the roles of another lncRNA in the risk model, AC083900.1 was identified as a prognostic Cuproptosis-associated lncRNA, which was upregulated in different osteosarcoma cell lines as well as the prediction of metastasis^57^. The remaining two AASRs, AC093627.22, and AP000802.1, were first reported in OS.

There are also several limitations in our study. First, although the prognostic signature exhibits good performance in predicting the overall survival of OS patients, due to the lack of lncRNA transcriptome data, the signature needs to be further verified in additional external datasets or clinical samples. Second, few studies have been designed to reveal the roles of these AASRs in OS. Future research is warranted to explore the molecular biological functions and mechanisms of the other AASRs in OS senescence, tumorigenesis and progression. Last, in vivo experiments are needed to further verify the molecular mechanism of UNC5B-AS1 in OS.

## Conclusion

In conclusion, we identified a 5 AASR-based signature that could well predict the overall survival of OS patients. We also experimentally demonstrated that UNC5B-AS1 might inhibit OS cell senescence and apoptosis, thereby promoting the development and progression of OS.

### Supplementary Information

Supplementary Information 1.

Supplementary Information 2.

## Data Availability

The analyzed data sets generated during the present study are obtained from the TCGA database (https://portal.gdc.cancer.gov/), and the human AAGs were obtained from Human Aging Genomic Resources (HAGR) database (http://genomics.senescence.info/).
