# Supplementary material for: A novel aging-associated lncRNA signature for predicting prognosis in osteosarcoma
Source: Sci Rep. 2024 Jan 16;14:1386. doi: 10.1038/s41598-024-51732-1 (PMC10791644; doi:10.1038/s41598-024-51732-1)
Supplement: Supplementary file 1 — Supplementary Information 1. [file 41598_2024_51732_MOESM1_ESM.pdf]

Supplementary Figure 1. All original blots of p21, PCNA and β-tubulin

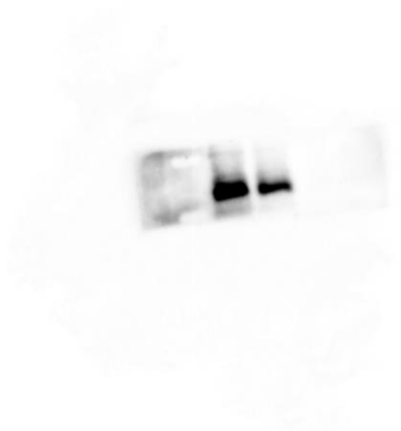

p21 exposure 1 (left: UNC5B-AS1 siRNA, right: control siRNA)

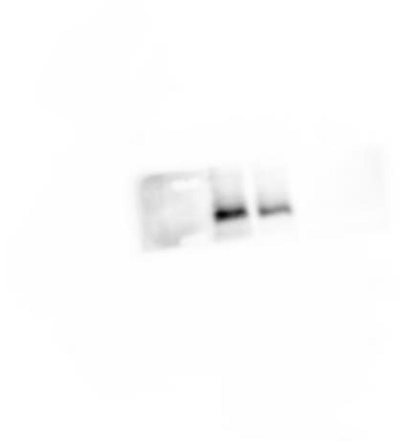

p21 exposure 2 (left: UNC5B-AS1 siRNA, right: control siRNA)

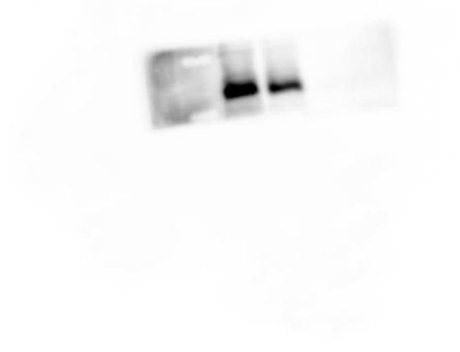

p21 exposure 3 (left: UNC5B-AS1 siRNA, right: control siRNA)

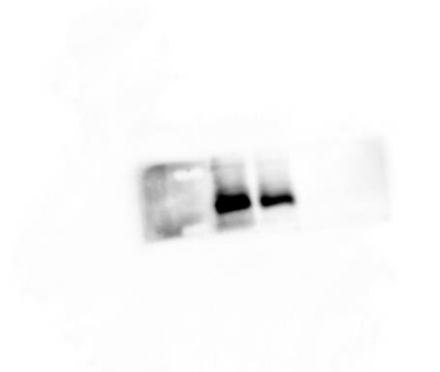

p21 exposure 4 (left: UNC5B-AS1 siRNA, right: control siRNA)

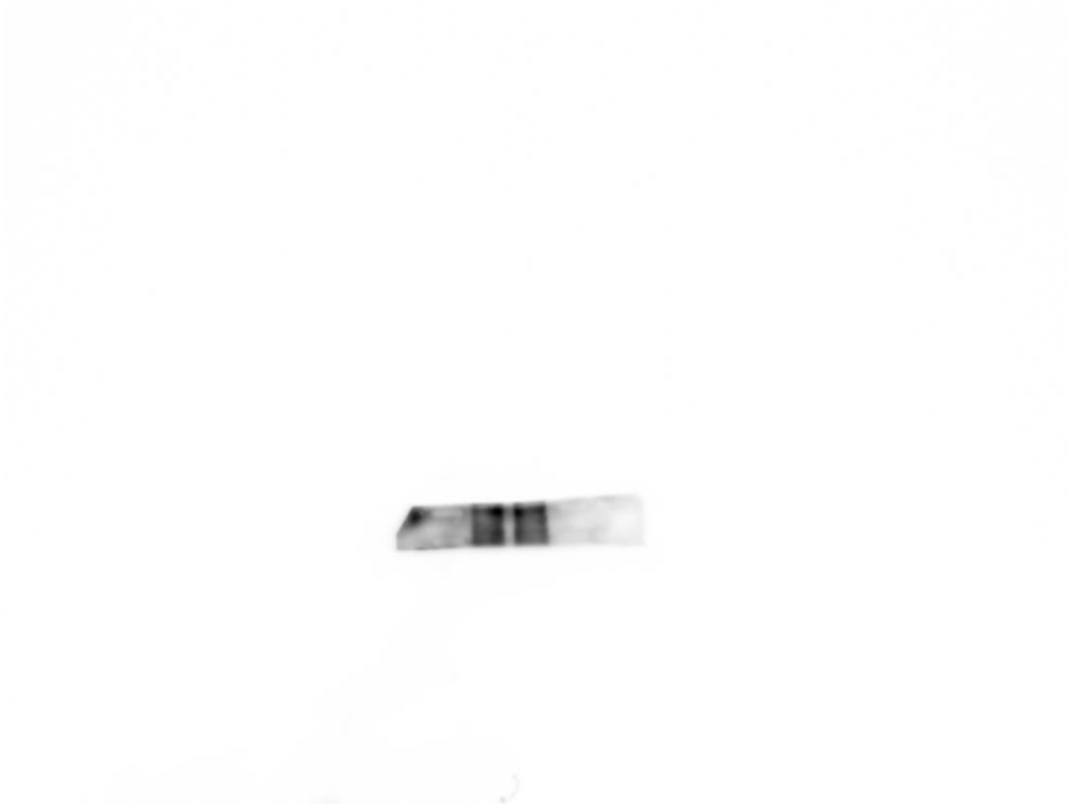

PCNA exposure 1 (left: UNC5B-AS1 siRNA, right: control siRNA)

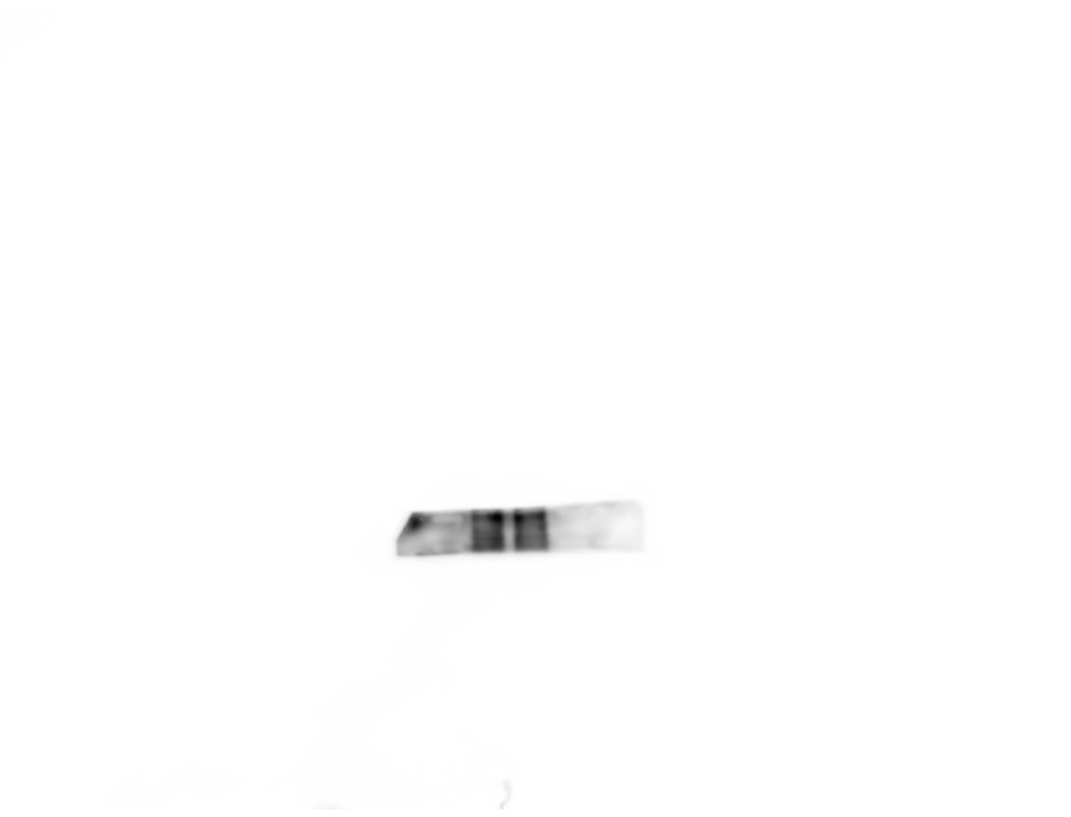

PCNA exposure 2 (left: UNC5B-AS1 siRNA, right: control siRNA)

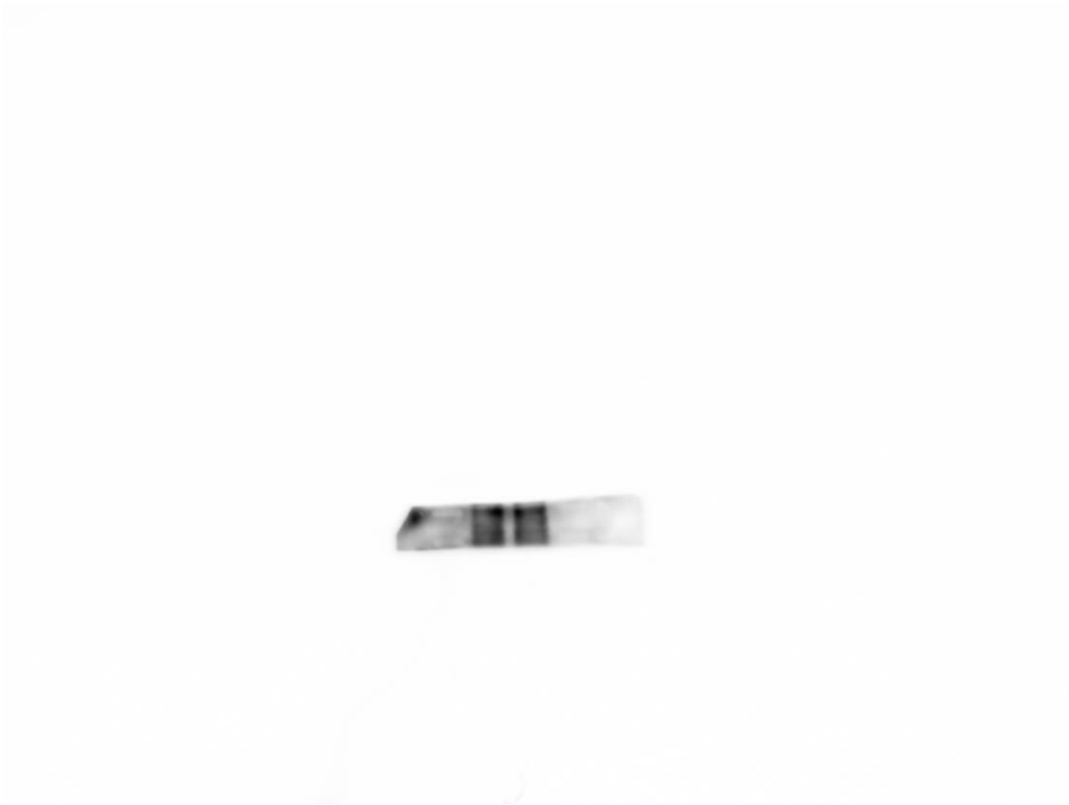

PCNA exposure 3 (left: UNC5B-AS1 siRNA, right: control siRNA)

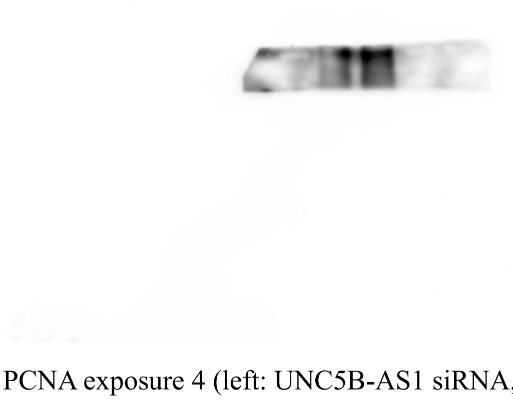

PCNA exposure 4 (left: UNC5B-AS1 siRNA, right: control siRNA)

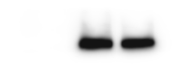

β-tubulin exposure 1 (left: UNC5B-AS1 siRNA, right: control siRNA)

β-tubulin exposure 2 (left: UNC5B-AS1 siRNA, right: control siRNA)

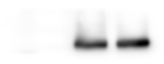

β-tubulin exposure 3 (left: UNC5B-AS1 siRNA, right: control siRNA)
