# Supplementary material for: A novel aging-associated lncRNA signature for predicting prognosis in osteosarcoma
Source: Sci Rep. 2024 Jan 16;14:1386. doi: 10.1038/s41598-024-51732-1 (PMC10791644; doi:10.1038/s41598-024-51732-1)
Supplement: Supplementary file 2 — Supplementary Information 2. [file 41598_2024_51732_MOESM2_ESM.docx]

| Column Header | characteristic | Number |
| --- | --- | --- |
| Gender | Male | 46 |
|  | Female | 37 |
| Age | <=14 | 38 |
|  | >14 | 45 |
| Vital Status | Alive | 56 |
|  | Dead | 27 |
| Disease at diagnosis | Non-metastatic | 63 |
|  | Metastatic | 21 |
| Specific tumor site | Arm | 12 |
|  | Leg | 69 |
|  | Pelvis | 1 |
|  | Ilium | 1 |

Supplementary Table 1. The clinical information of Osteosarcoma patients
